# Individual differences in brain structure and self-reported empathy in children

**DOI:** 10.3758/s13415-022-00993-2

**Published:** 2022-03-25

**Authors:** Katherine O. Bray, Elena Pozzi, Nandita Vijayakumar, Sally Richmond, Camille Deane, Christos Pantelis, Vicki Anderson, Sarah Whittle

**Affiliations:** 1 Melbourne Neuropsychiatry Centre (MNC), Department of Psychiatry, The University of Melbourne & Melbourne Health, Melbourne, Australia; 2 Melbourne School of Psychological Sciences, University of Melbourne, Melbourne, Australia; 3 School of Psychology, Deakin University, Melbourne, Australia; 4 Turner Institute for Brain and Mental Health, School of Psychological Sciences, Monash University, Melbourne, Australia; 5 Murdoch Children’s Research Centre, Melbourne, Australia

**Keywords:** Affective empathy, Cognitive empathy, Brain structure, Voxel-based morphometry, Late childhood

## Abstract

Empathy refers to the understanding and sharing of others’ emotions and comprises cognitive and affective components. Empathy is important for social functioning, and alterations in empathy have been demonstrated in many developmental or psychiatric disorders. While several studies have examined associations between empathy and brain structure in adults, few have investigated this relationship in children. Investigating associations between empathy and brain structure during childhood will help us to develop a deeper understanding of the neural correlates of empathy across the lifespan. A total of 125 children (66 females, mean age 10 years) underwent magnetic resonance imaging brain scans. Grey matter volume and cortical thickness from structural images were examined using the Computational Anatomy Toolbox (CAT12) within Statistical Parametric Mapping (SPM12) software. Children completed questionnaire measures of empathy (cognitive empathy, affective empathy: affective sharing, empathic concern, and empathic distress). In hypothesised region of interest analyses, individual differences in affective and cognitive empathy were related to grey matter volume in the insula and the precuneus. Although these relationships were of similar strength to those found in previous research, they did not survive correction for the total number of models computed. While no significant findings were detected between grey matter volume and empathy in exploratory whole-brain analysis, associations were found between cortical thickness and empathic concern in the right precentral gyrus. This study provides preliminary evidence that individual differences in self-reported empathy in children may be related to aspects of brain structure. Findings highlight the need for more research investigating the neurobiological correlates of empathy in children.

**Supplementary Information:**

The online version contains supplementary material available at 10.3758/s13415-022-00993-2.

Empathy is a multidimensional construct (Davis, 1983), which describes the ability to understand and share the emotions of other people with whom we interact (Shamay-Tsoory, 2011). Empathic abilities are established at an early age, are refined across development (Tousignant et al., 2017), and are crucial for social functioning (Cliffordson, 2002; Eisenberg et al., 1996) and mental health (Decety & Moriguchi, 2007; Farrow & Woodruff, 2007). As such, understanding the neural correlates of empathy across the lifespan is of importance for better understanding the mechanisms driving social functioning and mental illness.

Importantly, different components of empathy develop along differing trajectories, and are differentially related to outcomes. Affective sharing (i.e., “I *feel* what you feel”; Shamay-Tsoory et al., 2009), the capacity to share the same emotion as another (Decety & Moriguchi, 2007), develops early in life (Zahn-Waxler & Van Hulle, 2012) and has been found to be selectively impaired in psychopathy (Wai & Tiliopoulos, 2012). Cognitive empathy, the ability to infer and understand another’s mental state (the thoughts and feelings of another) and use this information to explain and predict human behaviour (i.e., “I *understand* what you feel”; Shamay-Tsoory et al., 2009), develops later (Devine & Hughes, 2016), and impairments have been linked to autism spectrum disorder (Quinde-Zlibut et al., 2021). Two additional affective empathy components are: empathic concern, which describes experiencing feelings of sympathy, compassion, or concern for another person in distress (Davis, 1983), and empathic distress, also known as personal distress, which refers to the dispositional tendency to experience “feelings of discomfort, uneasiness and distress when exposed to the distress of others” (Davis et al., 1994, p. 370). Empathic concern and distress develop differently and are known to be differentially associated with costly altruism (FeldmanHall et al., 2015), and emotion regulation (Eisenberg et al., 2010).

In adults, there have been several studies and meta-analyses that have investigated the neural correlates of empathy, with cognitive versus affective empathy suggested to be associated with different neurobiology. Cognitive empathy has been linked to default mode network regions such as the medial prefrontal cortex (mPFC), the precuneus/posterior cingulate cortex, the posterior superior temporal sulcus (STS)/temporoparietal junction (TPJ), and the temporal poles (Frith & Frith, 2006; meta-analyses by Molenberghs et al., 2016; Schurz et al., 2014). The ventral mPFC, precuneus and TPJ are considered to represent the core of the cognitive empathy network (Atique et al., 2011).

Conversely, affective empathy, particularly affective sharing, has been linked to the anterior insula (AI) extending to the inferior frontal gyrus (IFG) and the anterior cingulate cortex (ACC)/midcingulate cortex (MCC), particularly the dorsal ACC/anterior MCC. These core regions are involved in affective sharing of various emotions or states, such as disgust, pleasant feelings, or physical and emotional pain (Bernhardt & Singer, 2012; Fan et al., 2011; Lamm et al., 2011). The insular cortex is important in representing and integrating internal states and emotions, while the ACC has been described as the motivational and action related counterpart (Bernhardt et al., 2014).

The neural correlates of other specific components of affective empathy: empathic concern, and empathic distress have been preliminarily investigated. Several task-based functional magnetic resonance imaging (fMRI) studies in adults have found overlapping neural correlates with affective sharing; however, there also are unique correlates (e.g., ventral striatum; Kanske et al., 2015; Klimecki et al., 2014; Lamm et al., 2011), not surprising given their distinct behavioral associations.

Our understanding of the neural correlates of empathy has been largely based on task-based fMRI. For researchers investigating the neural correlates of individual differences in trait empathy, measures of brain structure are of particular interest given high reliability (Madan & Kensinger, 2017). From the small number of existing structural MRI studies in adults, the differentiation between affective and cognitive empathy appears less apparent as compared to functional studies. Several studies report structural differences in the AI related to affective empathy components (affective sharing or empathic concern) (Banissy et al., 2012; Eres et al., 2015; Hou et al., 2017; Patil et al., 2017; Valk et al., 2017; Yue et al., 2016), whereas differences in cingulate and frontal regions have been primarily implicated in cognitive empathy (Banissy et al., 2012; Eres et al., 2015; Uribe et al., 2019). Most studies found higher empathy to be related to increased grey matter volume (GMV)/cortical thickness; however, two studies (Banissy et al., 2012; Luo et al., 2018) found reduced GMV associated with increased empathy.

Furthermore, very few studies have investigated the neural correlates of empathy in children, which is an important endeavor given marked developmental changes in empathy. Basic empathy components emerge in children from a young age. The affective component is the first to come online, and rudimentary forms of emotion contagion and empathic distress are present in newborns (Zahn-Waxler & Van Hulle, 2012). Around the age of 1, caring behaviors start to occur (Zahn-Waxler et al., 1992). The ability to detect distress in others improves with age, and prosocial responses increase, becoming more varied and specific (Vaish et al., 2009; Zahn-Waxler & Van Hulle, 2012). The cognitive component lags behind the affective. Obvious milestones include the acquisition of first-order false belief, with most children passing explicit tests around age 5 to 6 years (O’Reilly & Peterson, 2015). Researchers have demonstrated that these abilities continue to develop throughout middle childhood and even into adolescence (Devine & Hughes, 2016; Dumontheil et al., 2010), allowing the mastering of the subtleties of understanding emotions and predicting social behavior. Similar to adults, the different components of empathy are differentially related to outcomes during childhood. For example, within the same sample being examined in the current study, affective components including affective sharing and empathic distress were associated with anxiety symptoms in children, whereas cognitive empathy was not (Bray et al., 2021). In other work, empathic concern was related to prosocial behavior in children, whilst empathic distress was negatively related or unrelated to prosocial behavior (Eisenberg et al., 2010).

Regarding existing MRI literature in children, some fMRI studies, albeit with small samples, report similar activation between children and adults during cognitive empathy tasks, namely recruitment of the mPFC and the STS/TPJ (Ohnishi et al., 2004; Saxe et al., 2009). In contrast, Decety and Michalska (2010) describe several differences in brain activity between children and adults viewing others in pain. Children had lower activity in the dorsolateral PFC and IFG, more medial orbitofrontal cortex activation, and stronger activation in the posterior compared to AI. Saxe et al. (2009) found that in children aged 6-11 years, the younger children recruited the right TPJ equally when thinking about other people’s mental states (cognitive empathy) and physical facts about people, but the older children only recruited this region for mental states, suggesting specialisation of this region during development.

There is a paucity of structural literature that focuses on empathy in typically developing children. Only a few studies have examined structural individual differences in children not impacted by a specific mental health or developmental disorder, and they have not examined different components of empathy. Stern et al. (2019) found that larger bilateral hippocampal volume was associated with greater empathic responding (expression of comforting behavior in response to experimenter distress) in boys aged 4-8 years. Sassa et al. (2012) examined the associations between general empathy (scores on the Empathising Quotient questionnaire) and GMV in a group of 5- to 15-year-old children. They found that empathy was positively associated with GMV in the left fronto-opercular and superior temporal cortices (including the precentral gyrus, the IFG, the STS, and the insula).

The current study was designed to build on the small number of studies investigating associations between empathy and grey matter structure during childhood, using multidimensional empathy measurement and both hypothesis-driven region of interest (ROI) and exploratory whole brain approaches. GMV was investigated in primary analyses to allow comparison with findings from most existing studies in adults. We also examined cortical thickness in whole brain analyses. While studies demonstrate reductions in both GMV and cortical thickness (i.e., thinning) between childhood (preschool onwards) and adulthood (Tamnes et al., 2017; Vijayakumar et al., 2018), these brain measures have different developmental trajectories (volume is a combination of thickness and surface area) and should be examined separately. The period of late childhood is when key social-emotional skills are developing readily within a context of social change (e.g., becoming less dependent on the family unit) (Duong & Bradshaw, 2017; Franco & Levitt, 1998). Children already have in place the fundamental components of empathy, and have not yet entered adolescence where substantial brain change occurs (Foulkes & Blakemore, 2018). For the current study, we chose to investigate children within late childhood, averaging 10 years old.

Given the lack of structural MRI studies in children, we based hypotheses on prior structural studies in adults and meta-analyses of fMRI studies. We hypothesised that affective empathy (affective sharing) would be associated with volume and/or cortical thickness of the AI/IFG, aMCC/dACC, and the supplementary motor area (SMA), while cognitive empathy would be related to volume of the precuneus, TPJ, and mPFC. We did not predict a specific direction of this relationship (increased versus decreased volume/cortical thickness) given that the associations between brain structure and empathy in adults may not necessarily extend to children, where the developmental context involves processes such as cortical reduction. In addition to cognitive empathy and affective sharing, we investigated the structural correlates of other components of empathy, empathic concern, and distress. Due to there being less research to date on the neural correlates of empathic concern and empathic distress, analyses for these components were exploratory. However, it was hypothesised that these components may share some overlap with the affective sharing components but also may have their own unique correlates (based on Kanske et al., 2015; Klimecki et al., 2014).

## Methods

### Design

The current study used a subset of data from the Families and Childhood Transitions Study (FACTS), a longitudinal, community-based cohort study. The larger study consisted of two waves of data collection, approximately 18 months apart (Simmons et al., 2017). Ethical approval was granted by the University of Melbourne Human Research Ethics Office (#1339904). The current study used the data collected from the second wave of the project when both empathy measures and structural MRI brain images were collected (empathy was not assessed at baseline).

### Participants

A total of 125 children (*M* age = 10 years [*SD* = 4 months, range = 9 years, 5 months – 10 years 10 months], 66 females [53%]) participated in the wave two assessment and completed all relevant measures for this analysis (out of a total of 142 children that participated in wave two of the study). Children at wave one were screened and excluded if they had significant motor or sensory impairments, developmental or intellectual disorders, neurological conditions, history of head trauma/loss of consciousness, claustrophobia, presence/likelihood of nonremovable ferrous metals in their body, or were taking regular psychoactive or steroid medications. As the larger study aimed to maximize variation in socioeconomic status, recruitment (occurring mostly through schools) targeted metropolitan areas classified within the lower tertile of socioeconomic disadvantage (Pink, 2011). This targeting ensured that even families from lower socioeconomic areas were well represented across both waves of the study. Race and ethnicity were self-reported by participants’ parents. The majority were white (71%), followed by Asian (11%) or other (12%). Most participants were Australian (62%), 22% had mixed Australian with European or Asian heritage, 5% Asian heritage, 2% European, 6% other. Missing: 1 participant for neighborhood advantage and 6 participants for race/ethnicity.

### Measures

#### Empathy Measures

See *Supplementary Materials* for the empathy questionnaire presented to participants. For all empathy measures, higher scores indicate higher self-report of empathy.

##### Adolescent Measure of Empathy and Sympathy (AMES)

Cognitive empathy, affective empathy (i.e., affective sharing), and sympathy (i.e., empathic concern) were measured by a self-report questionnaire measure, the Adolescent Measure of Empathy and Sympathy (AMES; Vossen et al., 2015). Each subscale contains four items, for a total of 12 items, rated on a 5-point Likert scale. An example of an item measuring affective sharing is “when a friend is angry, I feel angry too.” The other feelings addressed by the affective sharing subscale are sadness, fear, and nervousness. An example of an item measuring cognitive empathy is “I can easily tell how others are feeling.” An example of an item measuring empathic concern is “I feel sorry for someone who is treated unfairly.” This measure has been validated in 10- to 15-year-olds and has robust psychometrics, including satisfactory internal consistency and test-retest reliability over 2 weeks (Vossen et al., 2015). The cognitive empathy and empathic concern subscales were validated against the perspective taking and empathic concern subscales of a well-used adult empathy self-report measure (the Interpersonal Reactivity Index; Davis, 1983).

##### Empathic Responsiveness Questionnaire, Empathic distress subscale

Empathic distress was measured using the Empathic Responsiveness Questionnaire (ERQ: Olweus & Endresen, 1998). The empathic distress subscale has three items, ranked on a Likert scale of 1 to 6, and is appropriate for 9- and 10-year-olds. While the original measure had four items, we combined two items with very minor differences (“When I see a girl who is distressed I sometimes feel like crying” and “When I see a boy who is distressed I sometimes feel like crying”) into one item (“When I see a girl/boy who is distressed I sometimes feel like crying”). Previous studies have found the ERQ scale to be internally consistent (Manger et al., 2001; Nickerson et al., 2009)

#### Scoring and missing data

Subscales were created from the sum of individual items. If at least 70% of the subscale items were present, then the subscale was calculated for the participant. Missing items in subscales with more than 70% of items present were imputed using the item average (three participants were missing one item each). Two participants were missing the empathic distress subscale but were included for the other empathy component analyses.

### Procedure

Children attended assessments with their parent, where they completed the MRI scan, questionnaires, and tasks.

#### MRI Assessment Procedures

Child participants were scanned using a Siemens 3.0 Tesla TIM Trio scanner (Siemens, Erlangen, Germany) at The Royal Children’s Hospital, Melbourne. Participants lay supine with their head supported in a 32-channel head coil. After familiarization with the scanner environment using a simulator (“mock scan”), participants underwent structural and functional scanning of the brain. Children were given the option of having one parent present in the scanner room with them. The MRI assessment lasted less than 1 hour. Children chose a movie to be played during the structural sequences.

#### Image Acquisition – Structural Scan

T1-weighted images were acquired with motion correction (MPRAGE MoCo, repetition time = 2,530 msec; echo time1 = 1.74 msec, echo time2 = 3.6 msec, echo time3 = 5.46 msec, echo time4 = 7.32 msec; flip angle = 7 , field of view = 256 × 256 mm^2^); 176 contiguous, 1.0-mm thick slices were produced (voxel dimensions = 1.0 mm^3^). The sequence duration was 5:19 minutes.

#### VBM Analysis

Voxel-based morphometry (VBM) is an automated method which examines structural MR images of the brain and conducts a voxel-wise comparison of the local concentration of grey matter (Ashburner & Friston, 2000). The structural MRI data were processed using Statistical Parametric Mapping (SPM) 12 software (version 7219, http://www.fil.ion.ucl.ac.uk/spm/software/spm12) and the Computational Anatomy Toolbox (CAT12 version 12.3 (1310); http://dbm.neuro.uni-jena.de/cat/; Gaser & Dahnke, 2016) and implemented using Matlab. CAT12 calculated volumes are robust and have been shown to be close to a simulated ground truth (Fillmer et al., 2018). The CAT12 toolbox is highly comparable to Freesurfer in regards to estimating cortical thickness and is capable of doing so in healthy and neurological populations, it is fast and reliable and has excellent test-retest scores. (Righart et al., 2017; Seiger et al., 2018).

All images were manually reoriented within the SPM software (anterior commissure set to be the “origin”; coordinates 0,0,0), and pitch was adjusted so that the anterior commissure – posterior commissure line was horizontal. If the yaw or roll of images were visibly asymmetrical it also was adjusted. Pre-processing consisted of mostly standard VBM processing procedures implemented in CAT12, described in the manual (http://dbm.neuro.uni-jena.de/cat12/CAT12-Manual.pdf). The batch editor was used in SPM12 and the batch scripts are available here: https://github.com/kbkatebray/empathy_VBM. In brief, the steps were; T1 images were normalized to a template space, and segmented into tissue types (grey and white matter and cerebrospinal fluid), estimation of total intracranial volume (used as a covariate in later modelling), and smoothing (the GM images were smoothed with the recommended 8 mm full-width at half-maximum (FWHM) smoothing kernel).

Two changes were made to the default settings described in the CAT12 manual. The first was using customized tissue probability maps (TPMs), and the second was using a customized DARTEL template. These changes were both made due to using a pediatric sample, as recommended by the manual. Reasons for using customized TPMs and templates are highlighted in Wilke et al. (2017). The age of participants contributing to the DARTEL priors contained in SPM12 is 48.6 ± 16.4 years. Brain changes as part of normal development mean that standard adult priors may not be appropriate for our sample.

##### Customized TPMs

The Cerebromatic (COM) Toolbox (the updated version of the Template-O-Matic [TOM] Toolbox suggested by the CAT12 manual) was used to create age and sex matched TPMs (https://www.medizin.uni-tuebingen.de/kinder/en/research/neuroimaging/software/cerebromatic-toolbox/). The COM Toolbox uses statistically generated regression parameters from a sample of 1,914 participants between the ages of 13 months and 75 years. Children’s images were taken from two datasets; the Study of Normal Brain Development conducted by the US National Institute of Health and the Cincinnati MR Imaging of Neurodevelopment study. The COM toolbox has been updated (from TOM8) to use a more flexible approach called multivariate adaptive regression splines (Wilke et al., 2017).

The other change to default settings was the creation of a customized DARTEL template, which is also recommended for pediatric data. Our template was made within the CAT12 toolbox and used the customized TPMs made in the COM Toolbox. The six-step DARTEL procedure uses an iterative non-linear registration approach and is explained in more depth in Wilke (2018).

#### Surface-based morphometry (SBM) analysis – Cortical thickness

Projection-based thickness is calculated by using tissue segmentation to estimate white matter distance and project the local maxima to other grey matter voxels by using a neighbor relationship described by the white matter distance (Dahnke et al., 2013). Pre-processing consisted of standard SBM processing procedures implemented in CAT12, described in the manual (http://dbm.neuro.uni-jena.de/cat12/CAT12-Manual.pdf). The batch editor was used in SPM12 and the batch scripts are available here: https://github.com/kbkatebray/empathy_VBM. The steps undertaken were similar to the VBM analyses, plus several additional steps including estimating cortical thickness during segmentation and smoothing with a 15-mm, full-width at half-maximum (FWHM) smoothing kernel.

#### Quality Control

Images were manually inspected for motion, gross anatomical artifacts, and adequate whole-brain coverage during the manual reorientation process. In addition, internal quality control measures within the CAT12 toolbox were used. These measures have been demonstrated to be able to detect aspects of image quality impacting final structural measures of interest, above and beyond visual inspection (Gilmore et al., 2021).

Homogeneity of the sample was checked before smoothing within the CAT12 toolbox. Mean correlation (measures the homogeneity of the data and is a measure of image quality after pre-processing), weighted overall image quality (combines measurements of noise and spatial resolution of the images before pre-processing), and Mahalanobis distance (difference between two previously mentioned measures) were used. Analyses were re-run excluding three potential outliers identified in this process. Excluding these participants (see Supplementary Table S3 for results removing outliers) did not change any findings. After the second level model was specified, design orthogonality was checked and was not problematic (total intracranial volume [TIV] was independent from other parameters of interest).

#### Statistical Analysis

Full factorial models were specified in SPM12. Specifically, multiple regressions were used to examine the relationship between each of the empathy components separately (affective sharing, cognitive empathy, empathic concern, and empathic distress) and GMV or cortical thickness within SPM12. For volume, age, sex and TIV were entered as covariates in the model. Threshold masking with the recommended absolute value of 0.1 was used. For thickness, age and sex were entered as covariates in the model (TIV not recommended as a covariate, as it does not often correlate with surface measures such as cortical thickness, http://dbm.neuro.uni-jena.de/cat12/CAT12-Manual.pdf). Threshold masking was not used, as recommended, http://dbm.neuro.uni-jena.de/cat12/CAT12-Manual.pdf.

##### Region of interest selection and analysis (GMV)

For affective empathy and cognitive empathy, ROI analyses were performed using individual ROIs based on previous fMRI meta-analyses. Fan et al. (2011) was used for affective sharing (insula/inferior frontal gyrus [IFG; Brodman’s area (BA) 13/47], aMCC/dACC [BA 32], SMA [BA 6]) and Schurz et al. (2014) for cognitive empathy (precuneus [BA 7], superior temporal gyrus [BA 22, posterior TPJ], superior frontal gyrus [BA 9, mPFC]). Four mm spheres were created within WFU PickAtlas Toolbox (Maldjian et al., 2003), around the peak coordinates identified in the meta-analyses. Left and right equivalents were made for ROIs that did not have a lateral pair. See *Supplementary Materials* for details regarding the ROIs (Supplementary Table S1; Supplementary Fig. S1). A voxel-level threshold of *p* < 0.001 and a cluster-level threshold of *p* < 0.05 (family-wise error [FWE] corrected for multiple comparisons) were utilised. To apply the search within the area of the ROIs, small-volume correction was used within SPM. Additional correction was used to account for the numbers of models run: 12 seeds x 4 empathy measures = 48 models; adjusted *p*FWE < 0.001, critical α for cluster correction based on Bonferroni adjustment; Roiser et al., 2016). An alternative ROI approach was also pursued, using anatomical ROIs as opposed to coordinate-based ROIs. Please see *Supplementary Materials* for details.

##### Whole-brain analyses (GMV and cortical thickness)

Exploratory whole brain analyses (for volume and thickness) were run for each empathy component using a voxel level threshold of *p* < 0.001 and a cluster level threshold of *p* < 0.05 (family-wise error corrected for multiple comparisons).

A note on terminology: the product of the first-level VBM analysis in CAT12 is an image which is both “normalized” (“concentration” of grey matter; Good et al., 2001) and “modulated (“volume” of grey matter; Good et al., 2001; http://dbm.neuro.uni-jena.de/cat12/CAT12-Manual.pdf). This allowed examination of the relative differences in regional GMV across the sample; hence, we use the term GMV as opposed to others, such as density.

## Results

Descriptive Statistics calculated for the empathy measures are shown in Table 1.

**Table 1:** Means, standard deviations, range and internal consistency for the key measures used

Measure of interest	*N*	Mean	*SD*	Min-Max	Cronbach’s *α*
Affective sharing	125	9.52	3.84	4-20	0.86
Cognitive empathy	125	13.48	2.82	4-20	0.69
Empathic concern	125	16.54	2.38	9-20	0.60
Empathic distress	123	8.27	3.18	3-18	0.66

### ROI Analyses

**Fig. 1:**
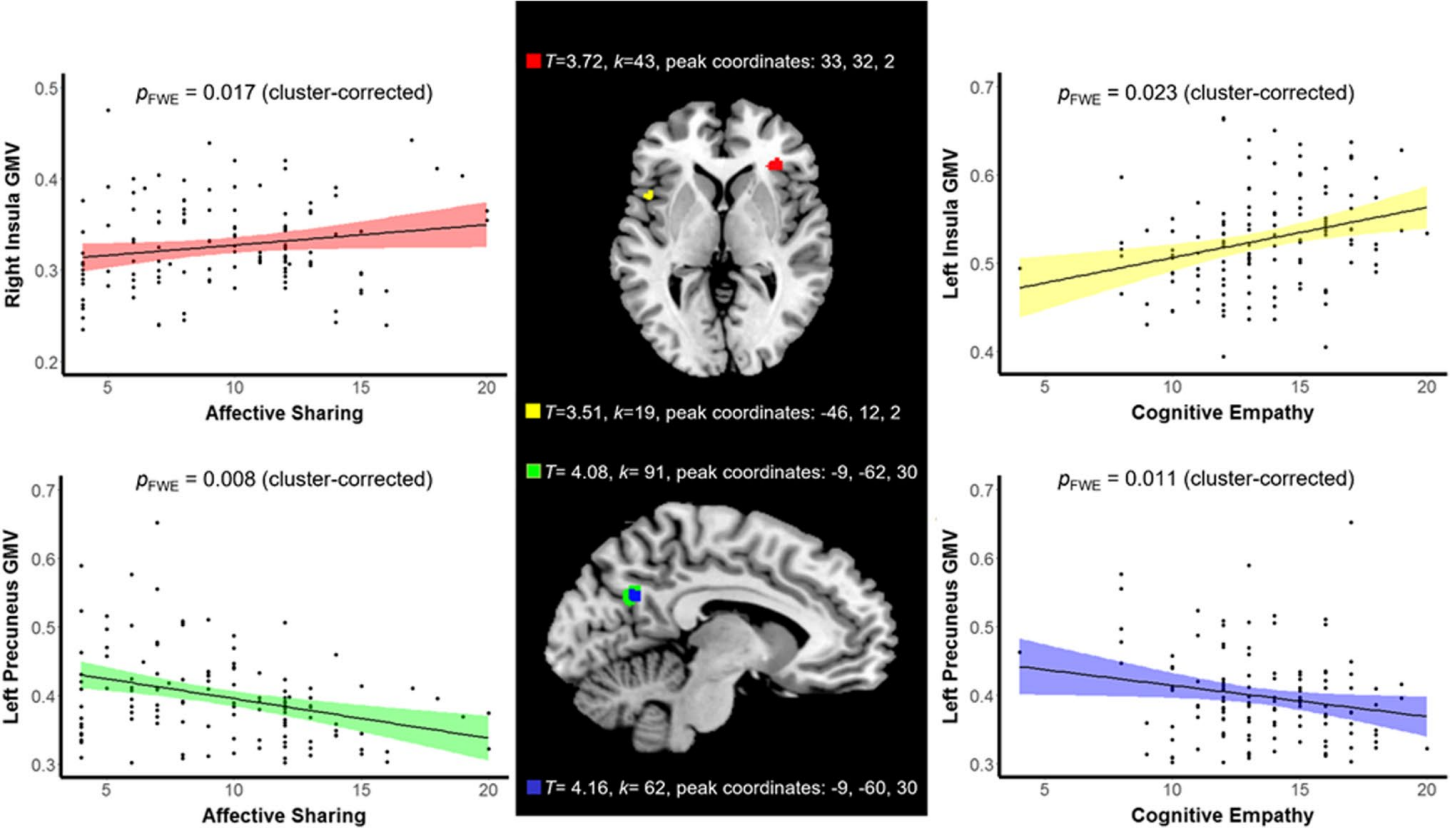
Higher self-reported affective sharing was related to increased GMV in the right AI, while higher self-reported cognitive empathy was related to increased GMV in the left AI (top half of figure). Higher self-reported affective sharing and cognitive empathy were both related to decreased GMV in the left precuneus (bottom half of figure). These findings did not survive correction for the total number of models computed. GMV displayed on scatterplot is for a 4-mm diameter sphere surrounding the peak within the significant cluster, presented for illustrative purposes. GMV, grey matter volume; AI, anterior insula; FWE, family-wise error

Affective sharing was positively related to right AI volume. Cognitive empathy was found to be positively related to left AI volume. Both cognitive and affective empathy were negatively related to left precuneus volume (see Table 2 and Fig. 1 for statistics and visualizations). These results did not withstand correction for the total number of models computed. The alternative anatomical ROI approach produced similar results (see *Supplementary Materials* for details).

**Table 2:** Statistics for the four significant findings in regions of interest

Empathy component	Association direction	Small volume correction area	Cluster-level *p*FWE-corrected	Cluster size (*k*)	*T*	Peak coordinates (MNI)
Affective sharing	Positive	Right IFG/AI	0.017	43	3.72	33, 32, 2
Cognitive empathy		Left IFG/AI	0.023	19	3.51	-46, 12, 2
Affective sharing	Negative	Left precuneus	0.008	91	4.08	-9, -62, 30
Cognitive empathy		Left precuneus	0.011	62	4.16	-9, 60, 30

*Note:* These findings do not withstand correction for the total number of models computed (adjusted α = 0.001). FWE, Family-wise error; IFG, inferior frontal gyrus; AI, anterior insula; MNI, Montreal Neurological Institute coordinate system

### Whole brain analyses (GMV and cortical thickness)

No clusters survived the cluster-level threshold of *p* < 0.05 (family-wise error corrected for multiple comparisons) at the level of the whole brain, for the relationship between any of the four empathy components and GMV.

**Fig. 2:**
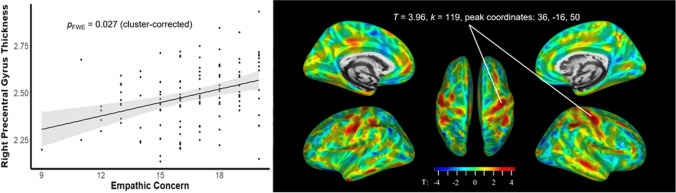
Higher self-reported empathic concern was related to increased cortical thickness (mm) in the right precentral gyrus. Thickness displayed on scatterplot is for the coordinates of the peak within the significant cluster. FWE, family-wise error.

Increased empathic concern ratings were related to increased cortical thickness within the right precentral gyrus (*T* = 3.96, *p*FWE = 0.027 (cluster-corrected), *k* = 119, peak coordinates: 36, -16, 50, see Fig. 2 for visualizations). The unthresholded statistical maps can be found on Neurovault (https://identifiers.org/neurovault.collection:9288). See Supplementary Table S4 and S5 for the uncorrected whole-brain results for GMV and cortical thickness.

## Discussion

The purpose of this study was to explore the structural neural underpinnings of empathy in children. A priori ROI analyses were performed for GMV, as well as more exploratory whole-brain analyses for both volume and cortical thickness. Four components of empathy were examined using self-report measures. This study found preliminary evidence for empathy-related individual differences in GMV comparable to some of the findings in previous adult literature (Eres et al., 2015); however, these findings did not withstand correction for the total numbers of models computed. Specifically, we found that higher self-reported affective sharing and cognitive empathy were related to increased right and left AI volume (respectively) and decreased left precuneus volume. The finding of increased affective empathy relating to increased AI volume also was found by Yue et al. (2016) and Eres et al. (2015). As described earlier, the AI is thought to represent internal states and has been implicated strongly in empathy (particularly affective empathy; Bernhardt & Singer, 2012; Fan et al., 2011; Lamm et al., 2011). The involvement of the precuneus in cognitive empathy has been established in fMRI meta-analyses (Molenberghs et al., 2016; Schurz et al., 2014). However, the only structural study to implicate the precuneus to date (Banissy et al., 2012) found higher empathic concern (an affective component of empathy) related to decreased precuneus volume. The current study found similar negative associations, however these were present for both cognitive and affective empathy.

One difference between the current ROI findings and previous literature is that there appeared to be no clear differentiation between the areas related to affective versus cognitive empathy. The insula and precuneus were implicated in both empathy types. The majority of the fMRI literature and some structural MRI studies (Eres et al., 2015) suggest that affective and cognitive empathy relate to different brain areas, with the insula involved in affective empathy, and the precuneus involved in cognitive empathy. Our findings support the possibility that children might not show the same pattern of differentiation as adults, although caution should be given to this potential explanation given that significant findings did not withstand correction for multiple comparisons. If replicated, the findings may suggest a lack of maturation or differentiation of neural subsystems subserving different empathy processes in childhood.

Alternatively, recent research may shed light on the lack of dissociation between affective and cognitive empathy we found in our study. Schurz et al. (2020) conducted a neuroimaging fMRI meta-analysis (in adults) investigating empathy and related sociocognitive and socioaffective processes. They created a clustering of meta-analytic results, to examine common underlying neurocognitive components engaged by different sociocognitive or affective tasks, including empathy tasks. The clustering found three groups of underlying processes; a cognitive cluster, an affective cluster and an “intermediate” cluster. The intermediate cluster was so named, because it was hypothesized to have contributions from *both* cognitive and affective processes. The brain areas implicated included traditional sociocognitive areas, such as the temporal lobes, temporoparietal cortex, and precuneus, and traditionally socioaffective areas, such as the insula and IFG. Tasks that fell into this intermediate cluster included reasoning about emotions, whereas tasks that were implicated in the cognitive-only cluster included reasoning about thoughts. Schurz et al. (2020) speculated that the intermediate cluster may relate to tasks often labelled as affective theory of mind or cognitive empathy, thus suggesting researchers may need to reevaluate certain measures of cognitive empathy and consider that they may draw upon both cognitive and affective processes simultaneously. Our measure of cognitive empathy captured reasoning about thoughts *and* emotions. These findings could potentially explain why we found *both* insula and precuneus brain structure related to cognitive empathy. Continued refinement of conceptualization and measurement of empathy in combination with data-driven approaches may help to clarify the underlying neural correlates of various empathy related processes.

At the level of the whole brain, no associations were found between empathy components and GMV. Regarding cortical thickness, we found that increased thickness in the right precentral gyrus related to increased empathic concern. This finding is consistent with some previous research. Sassa et al. (2012) also found associations between precentral gyrus structure and empathy in children; however, they found associations with volume rather than thickness, and their measure was one of general empathy (including both affective and cognitive components). Wildeboer et al. (2018) found associations between cortical thickness in the pre- and postcentral gyri and altruistic behaviour in the form of charitable giving in children (8 years old). Interestingly, altruistic behaviour has previously been linked to empathic concern scores (Batson, 2011). Several fMRI studies have also linked activity in the precentral gyrus with empathy. For example, receiving emotionally empathic comments after negative performance feedback elicited activation in several areas including the precentral gyrus (Seehausen et al., 2016). Greater neural activity in the precentral gyrus while detecting changes in a character’s mental and emotional state positively correlated with self-reported affective empathy (Hooker et al., 2010). Sensorimotor processes have been shown to be important in empathy for somatic pain via activations in the pre- and postcentral gyri (Riečanský & Lamm, 2019). However, these studies’ findings are not specific to empathic concern. Thijssen et al. (2015) reported negative associations between precentral gyrus cortical thickness and aggressive behavior in children, so it also may be speculated that this area may be involved in socioemotional behavior more generally. More studies are needed in children to establish whether this is a key brain region earlier in the development of empathy.

The less consistent associations found between empathy and brain structure may point toward the importance of investigating neural networks rather than single areas of the brain in isolation. Examining grey matter structural covariance may shed additional light, such as in the studies by Bernhardt et al. (2014) and Valk et al. (2017). They found no or few associations between empathy measures and the cortical thickness of individual brain regions but did find associations with the structural covariance between brain regions. For example, Valk et al. (2017) found that individual differences in cognitive empathy were related to covariance between temporoparietal and dorsomedial prefrontal areas, while conversely affective empathy was related to dorsal AI networks. It may be fruitful for future research to examine whether differences in empathy relate to structural covariance in children.

Several limitations should be considered in interpreting the current findings. There is little work that has investigated how self-report measures of empathy relate to empathic behaviour (assessed using empathy tasks). Recent research by Murphy and Lilienfeld (2019), focusing specifically on cognitive empathy, used meta-analytic techniques to demonstrate that self-report empathy did not relate to task-based measures of empathy. Future studies would benefit from using multiple empathy measures including those that are self-, parent-, or teacher-report, and objective tasks measuring different aspects of empathy. Our ROI-based findings did not survive stringent correction for the total numbers of models computed (based on the number of regions and empathy components). However, they are of similar strength to many of the ROI findings from previous adult studies. Finally, the children in our study were from a narrow age range and were only examined at one point in time. Longitudinal studies would be useful in this area for examining the trajectories of empathy and brain development, particularly in the context of normative linear and nonlinear decreases in cortical grey matter across childhood and adolescence.

## Conclusions

We found that both cognitive and affective empathy were associated with similar grey matter volumetric correlates in the AI and precuneus, partially consistent with previous findings in adults. Importantly, these associations did not withstand correction for total numbers of models computed, and as such, any interpretations should be made cautiously. We also found that increased thickness in the precentral gyrus was associated with empathic concern. Future research should use a range of empathy measures and examine data longitudinally.

## Supplementary information

ESM 1 (DOCX 593 kb)
