# Supplementary material for: Individual differences in brain structure and self-reported empathy in children
Source: Cogn Affect Behav Neurosci. 2022 Mar 25;22(5):1078–89. doi: 10.3758/s13415-022-00993-2 (PMC9458571; doi:10.3758/s13415-022-00993-2)
Supplement: Supplementary file 1 — (DOCX 593 kb) [file 13415_2022_993_MOESM1_ESM.docx]

# Supplementary Materials

## Empathy questionnaire as formatted for the current study

**Empathy Questionnaire**

We are going to ask you some questions about what you are like and how you normally behave. For each statement, please indicate how often this occurs.

|  |  | Never | Almost Never | Sometimes | Often | Always |
| --- | --- | --- | --- | --- | --- | --- |
| 1 | I can easily tell how others are feeling | 1 | 2 | 3 | 4 | 5 |
| 2 | I feel sorry for a friend who feels sad | 1 | 2 | 3 | 4 | 5 |
| 3 | I can often understand how people are feeling even before they tell me | 1 | 2 | 3 | 4 | 5 |
| 4 | I feel sorry for someone who is treated unfairly | 1 | 2 | 3 | 4 | 5 |
| 5 | When a friend is angry, I feel angry too | 1 | 2 | 3 | 4 | 5 |
| 6 | I am concerned for animals that are hurt | 1 | 2 | 3 | 4 | 5 |
| 7 | When my friend is sad, I become sad too | 1 | 2 | 3 | 4 | 5 |
| 8 | I can tell when a friend is angry even if he/she tries to hide it | 1 | 2 | 3 | 4 | 5 |
| 9 | When a friend is scared, I feel afraid | 1 | 2 | 3 | 4 | 5 |
| 10 | I can tell when someone acts happy, when they actually are not | 1 | 2 | 3 | 4 | 5 |
| 11 | I feel concerned for other people who are sick | 1 | 2 | 3 | 4 | 5 |
| 12 | When people around me are nervous, I become nervous too | 1 | 2 | 3 | 4 | 5 |

|  |  | Not true | Somewhat untrue | Rarely true | Somewhat true | Often true | | Always true |
| --- | --- | --- | --- | --- | --- | --- | --- | --- |
| 1 | It often makes me distressed when I see something sad on TV. | 1 | 2 | 3 | 4 | 5 | 6 | |
| 2 | Sometimes I feel a bit distressed when I read or hear about something | 1 | 2 | 3 | 4 | 5 | 6 | |
| 3 | When I see a girl/boy who is distressed I sometimes feel like crying. | 1 | 2 | 3 | 4 | 5 | 6 | |

Supplementary Table S1.

*Regions of interest used to probe affective and cognitive empathy components.*

| **Name** | **Coordinates (MNI: x,y,z)** |
| --- | --- |
| **Affective Empathy** |  |
| R insula/IFG^a^ | 38, 24 -2 |
| L insula/IFG^a^ | -42, 18, 0 |
| R aMCC/dACC^b^ | 2, 24, 38 |
| L aMCC/dACC^a^ | -2, 24, 38 |
| R SMA^a^ | 6, 8, 60 |
| L SMA^a^ | -4, 14, 54 |
| **Cognitive Empathy** |  |
| R precuneus^c^ | 4, -55, 34 |
| L precuneus^b^ | -4, -55, 34 |
| R superior temporal^c^ | 56, -56, 18 |
| L superior temporal^c^ | -53, -59, 20 |
| R superior frontal^b^ | 1, 56, 24 |
| L superior frontal^c^ | -1, 56, 24 |

*Note.* Abbreviations: right (R), left (L), inferior frontal gyrus (IFG), anterior mid-cingulate cortex (aMCC), dorsal anterior cingulate cortex (dACC), supplementary motor area (SMA), Montreal Neurological Institute (MNI). ^a^From Fan et al. (2011)^. b^ Left or right equivalent of the referenced regions of interest. ^c^From Schurz et al. (2014).


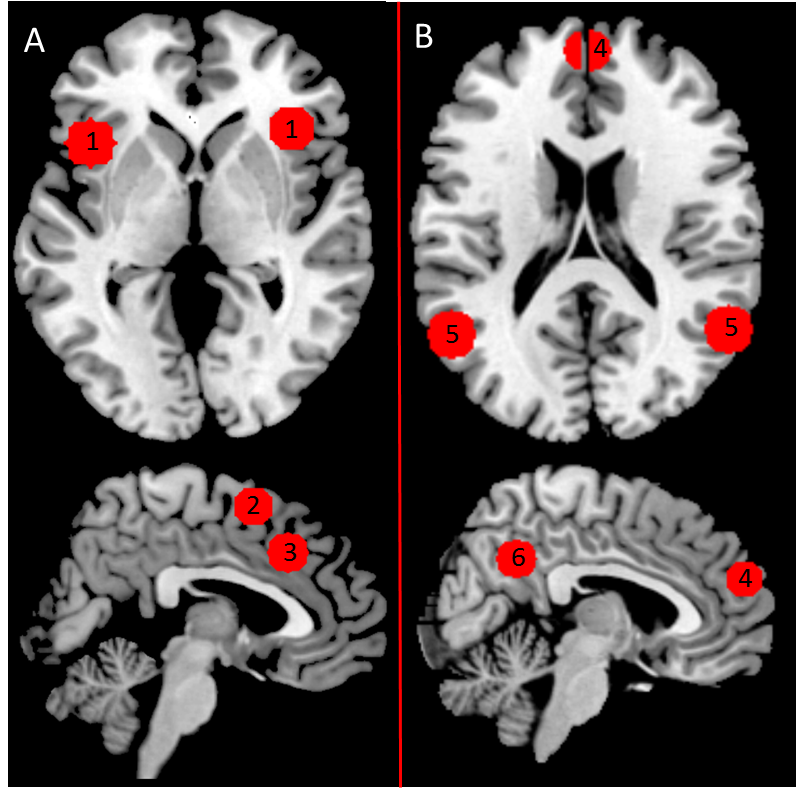


*Supplementary Figure S1*. Regions of interest (ROIs). A: ROIs relevant to affective sharing: insula/inferior frontal gyrus (1), supplementary motor area (2); anterior mid-cingulate cortex/dorsal anterior cingulate cortex (3), B: ROIs relevant to cognitive empathy: superior frontal (4), superior temporal (5), precuneus (6) (all regions right and left).

Supplementary Table S2

*Bivariate correlations for empathy variables (Pearson’s)*

|  | 1 | 2 | 3 | 4 |
| --- | --- | --- | --- | --- |
| 1. Affective Sharing | - |  |  |  |
| 2. Cognitive Empathy | .31** | - |  |  |
| 3. Empathic Concern | .32** | .51*** | - |  |
| 4. Empathic Distress | .50*** | .22* | .38*** | - |

*Note.* * *p* < 0.05; ** *p* < 0.01; *** *p* < 0.001. Not corrected for multiple comparisons.

Supplementary Table S3.

*Voxel-based morphometry results for the four significant findings at the level of the region of interest re-run while excluding the three participants identified in quality control measures.*

| Empathy component | Association direction | Small volume correction area | Cluster-level *p*FWE-corrected | Cluster size (*k*) | *T* | Peak coordinates |
| --- | --- | --- | --- | --- | --- | --- |
| Affective sharing | Positive | Right IFG/AI | 0.019 | 35 | 3.55 | 33, 32, 2 |
| Cognitive empathy |  | Left IFG/AI | 0.028 | 9 | 3.34 | -46, 12, 2 |
| Affective sharing | Negative | Left precuneus | 0.022 | 8 | 3.39 | -9, -62, 30 |
| Cognitive empathy |  | Left precuneus | 0.015 | 35 | 3.81 | -9, 60, 30 |

*Note.* These findings do not withstand correction for the total number of models computed. Abbreviations: Family-wise error (FWE), inferior frontal gyrus (IFG), anterior insula (AI).

Supplementary Table S4.

*Uncorrected results for whole-brain voxel-based morphometry (grey matter volume).*

| Empathy Component | Contrast | | | set-level | cluster-level | | | | | | peak-level | | | | | | mm | | |
| --- | --- | --- | --- | --- | --- | --- | --- | --- | --- | --- | --- | --- | --- | --- | --- | --- | --- | --- | --- |
|  |  | | | *p* | | *c* | *p*_FWE-corr_ | *q*_FDR-corr_ | *k*_E_ | *p*_uncorr_ | | *p*_FWE-corr_ | *q*_FDR-corr_ | *T* | (*Z*_E_) | *p*_uncorr_ | x | y | z |
| Affective sharing | *Positive* | | | 0.90 | | 5 | 0.23 | 0.17 | 425 | 0.03 | | 0.47 | 0.32 | 4.09 | 3.95 | 0.000 | 37.5 | -79.5 | -54 |
|  |  | | |  | |  | 0.85 | 0.59 | 117 | 0.24 | | 0.64 | 0.32 | 3.93 | 3.80 | 0.000 | 33 | 33 | 3 |
|  |  | | |  | |  | 0.99 | 0.94 | 25 | 0.60 | | 0.89 | 0.47 | 3.66 | 3.56 | 0.000 | 10.5 | -67.5 | -36 |
|  |  | | |  | |  | 1.00 | 0.94 | 2 | 0.91 | | 1.00 | 0.88 | 3.25 | 3.18 | 0.001 | 4.5 | 9 | 15 |
|  |  | | |  | |  | 1.00 | 0.94 | 1 | 0.94 | | 1.00 | 0.88 | 3.22 | 3.14 | 0.001 | -4.5 | 4.5 | 16.5 |
|  | *Negative* | | | 0.06 | | 13 | 0.11 | 0.18 | 598 | 0.01 | | 0.11 | 0.37 | 4.61 | 4.42 | 0.000 | 7.5 | -45 | 63 |
|  |  | | |  | |  |  |  |  |  | | 0.90 | 0.70 | 3.65 | 3.55 | 0.000 | 1.5 | -52.5 | 69 |
|  |  | | |  | |  |  |  |  |  | | 0.93 | 0.70 | 3.59 | 3.49 | 0.000 | 10.5 | -48 | 72 |
|  |  | | |  | |  | 0.75 | 0.79 | 158 | 0.17 | | 0.48 | 0.70 | 4.08 | 3.94 | 0.000 | -9 | -61.5 | 30 |
|  |  | | |  | |  | 0.77 | 0.79 | 150 | 0.18 | | 0.75 | 0.70 | 3.83 | 3.71 | 0.000 | -15 | 43.5 | 31.5 |
|  |  | | |  | |  | 0.95 | 0.82 | 67 | 0.37 | | 0.86 | 0.70 | 3.71 | 3.60 | 0.000 | -45 | -69 | -13.5 |
|  |  | | |  | |  | 0.98 | 0.82 | 43 | 0.48 | | 0.87 | 0.70 | 3.70 | 3.59 | 0.000 | -15 | -81 | 0 |
|  |  | | |  | |  | 0.97 | 0.82 | 50 | 0.44 | | 0.94 | 0.70 | 3.59 | 3.49 | 0.000 | 25.5 | 30 | -25.5 |
|  |  | | |  | |  |  |  |  |  | | 1.00 | 0.88 | 3.26 | 3.19 | 0.001 | 31.5 | 37.5 | -22.5 |
|  |  | | |  | |  | 0.98 | 0.82 | 39 | 0.50 | | 0.97 | 0.76 | 3.48 | 3.39 | 0.000 | 7.5 | -58.5 | 60 |
|  |  | | |  | |  | 1.00 | 0.94 | 14 | 0.70 | | 0.98 | 0.76 | 3.47 | 3.38 | 0.000 | -43.5 | -52.5 | 12 |
|  |  | | |  | |  | 0.98 | 0.82 | 38 | 0.51 | | 0.99 | 0.86 | 3.36 | 3.28 | 0.001 | 43.5 | -42 | 69 |
|  |  | | |  | |  | 1.00 | 0.94 | 1 | 0.94 | | 1.00 | 0.88 | 3.32 | 3.24 | 0.001 | -60 | -69 | -16.5 |
|  |  | | |  | |  | 1.00 | 0.94 | 1 | 0.94 | | 1.00 | 0.88 | 3.27 | 3.19 | 0.001 | -61.5 | -67.5 | -15 |
|  |  | | |  | |  | 1.00 | 0.94 | 1 | 0.94 | | 1.00 | 0.92 | 3.21 | 3.14 | 0.001 | -45 | -46.5 | 46.5 |
|  |  | | |  | |  | 1.00 | 0.94 | 4 | 0.86 | | 1.00 | 0.92 | 3.21 | 3.14 | 0.001 | 39 | 48 | 40.5 |
| Cognitive empathy | *Positive* | | | 0.90 | | 5 | 0.97 | 0.80 | 51 | 0.44 | | 0.30 | 0.31 | 4.27 | 4.11 | 0.000 | -36 | 34.5 | 4.5 |
|  |  | | |  | |  | 0.85 | 0.80 | 116 | 0.24 | | 0.84 | 0.35 | 3.73 | 3.62 | 0.000 | 37.5 | -33 | 40.5 |
|  |  | | |  | |  | 0.99 | 0.80 | 20 | 0.64 | | 0.87 | 0.35 | 3.70 | 3.59 | 0.000 | -46.5 | -12 | 27 |
|  |  | | |  | |  | 0.99 | 0.80 | 25 | 0.60 | | 0.97 | 0.49 | 3.51 | 3.42 | 0.000 | -46.5 | 12 | 1.5 |
|  |  | | |  | |  | 1.00 | 0.91 | 2 | 0.91 | | 1.00 | 0.75 | 3.28 | 3.20 | 0.001 | -22.5 | -15 | 57 |
|  | *Negative* | | | 0.06 | | 13 | 0.06 | 0.10 | 736 | 0.01 | | 0.10 | 0.22 | 4.62 | 4.42 | 0.000 | -3 | -48 | 64.5 |
|  |  | | |  | |  |  |  |  |  | | 0.91 | 0.96 | 3.63 | 3.53 | 0.000 | -18 | -40.5 | 72 |
|  |  | | |  | |  | 0.93 | 0.94 | 78 | 0.33 | | 0.40 | 0.51 | 4.16 | 4.01 | 0.000 | -9 | -60 | 30 |
|  |  | | |  | |  | 0.96 | 0.94 | 60 | 0.40 | | 0.84 | 0.96 | 3.73 | 3.62 | 0.000 | -33 | 13.5 | 37.5 |
|  |  | | |  | |  |  |  |  |  | | 0.99 | 0.96 | 3.40 | 3.32 | 0.000 | -34.5 | 1.5 | 33 |
|  |  | | |  | |  | 0.98 | 0.94 | 39 | 0.50 | | 0.95 | 0.96 | 3.56 | 3.47 | 0.000 | -52.5 | -63 | 1.5 |
|  |  | | |  | |  | 0.96 | 0.94 | 58 | 0.41 | | 0.97 | 0.96 | 3.48 | 3.39 | 0.000 | -48 | -55.5 | 49.5 |
|  |  | | |  | |  | 0.99 | 0.94 | 23 | 0.62 | | 0.98 | 0.96 | 3.45 | 3.36 | 0.000 | -42 | -28.5 | 61.5 |
|  |  | | |  | |  | 1.00 | 0.94 | 3 | 0.88 | | 1.00 | 0.96 | 3.32 | 3.24 | 0.001 | -28.5 | -22.5 | 51 |
|  |  | | |  | |  | 0.99 | 0.94 | 19 | 0.65 | | 1.00 | 0.96 | 3.31 | 3.23 | 0.001 | 25.5 | -81 | -6 |
|  |  | | |  | |  | 1.00 | 0.94 | 2 | 0.91 | | 1.00 | 0.96 | 3.25 | 3.17 | 0.001 | 28.5 | -15 | -10.5 |
|  |  | | |  | |  | 1.00 | 0.94 | 1 | 0.94 | | 1.00 | 0.96 | 3.22 | 3.15 | 0.001 | -12 | -90 | 9 |
|  |  | | |  | |  | 1.00 | 0.94 | 5 | 0.84 | | 1.00 | 0.96 | 3.22 | 3.14 | 0.001 | 22.5 | -75 | -12 |
|  |  | | |  | |  | 1.00 | 0.94 | 1 | 0.94 | | 1.00 | 0.96 | 3.21 | 3.13 | 0.001 | -37.5 | -25.5 | 70.5 |
|  |  | | |  | |  | 1.00 | 0.94 | 2 | 0.91 | | 1.00 | 0.96 | 3.18 | 3.10 | 0.001 | -24 | -45 | 78 |
| Empathic Concern | *Positive* | | | 0.96 | | 4 | 0.92 | 0.54 | 82 | 0.32 | | 0.95 | 0.73 | 3.55 | 3.46 | 0.000 | 69 | 6 | 21 |
|  |  | | |  | |  | 0.99 | 0.63 | 21 | 0.63 | | 0.96 | 0.73 | 3.53 | 3.44 | 0.000 | -13.5 | -70.5 | -37.5 |
|  |  | | |  | |  | 0.96 | 0.54 | 59 | 0.40 | | 0.96 | 0.73 | 3.52 | 3.43 | 0.000 | -49.5 | -4.5 | 10.5 |
|  |  | | |  | |  | 0.95 | 0.54 | 66 | 0.37 | | 0.97 | 0.73 | 3.49 | 3.39 | 0.000 | -64.5 | 6 | 6 |
|  | *Negative* | | | 0.11 | | 12 | 0.21 | 0.35 | 449 | 0.03 | | 0.50 | 0.58 | 4.06 | 3.92 | 0.000 | 34.5 | -34.5 | -28.5 |
|  |  | | |  | |  |  |  |  |  | | 0.83 | 0.58 | 3.75 | 3.64 | 0.000 | 43.5 | -55.5 | -21 |
|  |  | | |  | |  |  |  |  |  | | 0.86 | 0.58 | 3.71 | 3.60 | 0.000 | 43.5 | -42 | -25.5 |
|  |  | | |  | |  | 0.96 | 0.94 | 62 | 0.39 | | 0.72 | 0.58 | 3.86 | 3.74 | 0.000 | -22.5 | 12 | -31.5 |
|  |  | | |  | |  | 0.98 | 0.94 | 36 | 0.52 | | 0.80 | 0.58 | 3.78 | 3.66 | 0.000 | 49.5 | -46.5 | 7.5 |
|  |  | | |  | |  | 0.99 | 0.94 | 35 | 0.53 | | 0.88 | 0.58 | 3.68 | 3.58 | 0.000 | -19.5 | 55.5 | -7.5 |
|  |  | | |  | |  | 1.00 | 0.94 | 10 | 0.76 | | 0.91 | 0.58 | 3.63 | 3.53 | 0.000 | -51 | -24 | 31.5 |
|  |  | | |  | |  | 0.96 | 0.94 | 59 | 0.40 | | 0.96 | 0.69 | 3.52 | 3.42 | 0.000 | -52.5 | 9 | -15 |
|  |  | | |  | |  | 1.00 | 0.94 | 14 | 0.71 | | 0.97 | 0.69 | 3.50 | 3.40 | 0.000 | -13.5 | 18 | 40.5 |
|  |  | | |  | |  | 1.00 | 0.94 | 10 | 0.76 | | 0.99 | 0.89 | 3.37 | 3.28 | 0.001 | 16.5 | -69 | 39 |
|  |  | | |  | |  | 1.00 | 0.94 | 7 | 0.80 | | 1.00 | 0.95 | 3.29 | 3.21 | 0.001 | 49.5 | -33 | -16.5 |
|  |  | | |  | |  | 1.00 | 0.94 | 4 | 0.86 | | 1.00 | 0.95 | 3.22 | 3.15 | 0.001 | 42 | 39 | 21 |
|  |  | | |  | |  | 1.00 | 0.94 | 9 | 0.77 | | 1.00 | 0.95 | 3.21 | 3.13 | 0.001 | 34.5 | -63 | -13.5 |
|  |  | | |  | |  | 1.00 | 0.94 | 1 | 0.94 | | 1.00 | 0.96 | 3.18 | 3.11 | 0.001 | -58.5 | 15 | -21 |
| Empathic Distress | *Positive* | | | 0.17 | | 11 | 0.74 | 0.94 | 161 | 0.17 | | 0.49 | 0.74 | 4.07 | 3.93 | 0.000 | 21 | -12 | -9 |
|  |  | | |  | |  | 0.91 | 0.94 | 90 | 0.30 | | 0.75 | 0.74 | 3.82 | 3.70 | 0.000 | 1.5 | -18 | -4.5 |
|  |  | | |  | |  | 0.99 | 0.94 | 33 | 0.54 | | 0.94 | 0.74 | 3.58 | 3.48 | 0.000 | 61.5 | -19.5 | 12 |
|  |  | | |  | |  | 0.98 | 0.94 | 40 | 0.50 | | 0.94 | 0.74 | 3.57 | 3.47 | 0.000 | 31.5 | 33 | 6 |
|  |  | | |  | |  | 0.66 | 0.94 | 195 | 0.14 | | 0.96 | 0.74 | 3.53 | 3.44 | 0.000 | 7.5 | -72 | -34.5 |
|  |  | | |  | |  | 0.97 | 0.94 | 53 | 0.43 | | 0.97 | 0.74 | 3.49 | 3.40 | 0.000 | -54 | -55.5 | -21 |
|  |  | | |  | |  |  |  |  |  | | 1.00 | 0.95 | 3.27 | 3.19 | 0.001 | -61.5 | -55.5 | -25.5 |
|  |  | | |  | |  | 0.99 | 0.94 | 24 | 0.61 | | 0.99 | 0.87 | 3.39 | 3.30 | 0.000 | 3 | -63 | -13.5 |
|  |  | | |  | |  | 1.00 | 0.94 | 2 | 0.91 | | 1.00 | 0.95 | 3.25 | 3.18 | 0.001 | -15 | 42 | 4.5 |
|  |  | | |  | |  | 1.00 | 0.94 | 5 | 0.84 | | 1.00 | 0.95 | 3.22 | 3.15 | 0.001 | -73.5 | -22.5 | 0 |
|  |  | | |  | |  | 1.00 | 0.94 | 4 | 0.86 | | 1.00 | 0.95 | 3.21 | 3.13 | 0.001 | 37.5 | -76.5 | -46.5 |
|  |  | | |  | |  | 1.00 | 0.94 | 1 | 0.94 | | 1.00 | 0.95 | 3.18 | 3.11 | 0.001 | 70.5 | 3 | 21 |
|  | *Negative* | | | 0.67 | | 7 | 0.62 | 0.43 | 209 | 0.12 | | 0.35 | 0.27 | 4.21 | 4.06 | 0.000 | -13.5 | 43.5 | -12 |
|  |  | | |  | |  | 0.44 | 0.43 | 289 | 0.07 | | 0.57 | 0.27 | 3.99 | 3.86 | 0.000 | 22.5 | 24 | -25.5 |
|  |  | | |  | |  |  |  |  |  | | 0.61 | 0.27 | 3.96 | 3.83 | 0.000 | 21 | 27 | -16.5 |
|  |  | | |  | |  | 1.00 | 0.91 | 13 | 0.72 | | 0.92 | 0.57 | 3.62 | 3.52 | 0.000 | -12 | 36 | 10.5 |
|  |  | | |  | |  | 0.98 | 0.91 | 42 | 0.49 | | 0.96 | 0.61 | 3.52 | 3.43 | 0.000 | -34.5 | 3 | 30 |
|  |  | | |  | |  | 1.00 | 0.91 | 6 | 0.82 | | 0.99 | 0.80 | 3.35 | 3.27 | 0.001 | -16.5 | -75 | 1.5 |
|  |  | | |  | |  | 1.00 | 0.91 | 13 | 0.72 | | 1.00 | 0.80 | 3.30 | 3.22 | 0.001 | 0 | 46.5 | -15 |
|  | |  |  | | |  | 1.00 | 0.91 | 2 | 0.91 | | 1.00 | 0.96 | 3.18 | 3.11 | 0.001 | 15 | -13.5 | 49.5 |

*Note.* Abbreviations: familywise error (FWE), correction (corr), false discovery rate (FDR).

Supplementary Table S5.

*Uncorrected results for whole-brain surface-based morphometry (cortical thickness).*

| Empathy Component |  | set-level | | | cluster-level | | | | peak-level | | | | | mm | | |
| --- | --- | --- | --- | --- | --- | --- | --- | --- | --- | --- | --- | --- | --- | --- | --- | --- |
|  | Contrast | | *p* | *c* | *p*_FWE-corr_ | *q*_FDR-corr_ | *k*_E_ | *p*_uncorr_ | *p*_FWE-corr_ | *q*_FDR-corr_ | *T* | (*Z*_E_) | *p*_uncorr_ | x | y | z |
| Affective sharing | *Positive* | | 0.66 | 2 | 0.73 | 0.89 | 15 | 0.57 | 0.73 | 0.73 | 3.36 | 3.28 | 0.001 | 5 | -58 | 38 |
|  |  | |  |  | 0.87 | 0.89 | 3 | 0.89 | 0.81 | 0.73 | 3.28 | 3.2 | 0.001 | -66 | -23 | 3 |
|  | *Negative* | | No suprathreshold clusters | | | | | | | | | | | | | |
| Cognitive empathy | *Positive* | | 0.90 | 1 | 0.82 | 0.77 | 7 | 0.77 | 0.70 | 0.54 | 3.39 | 3.31 | 0 | 26 | -16 | -32 |
|  | *Negative* | | 0.90 | 1 | 0.26 | 0.13 | 54 | 0.13 | 0.10 | 0.05 | 4.19 | 4.04 | 0 | 1 | 11 | 23 |
| Empathic Concern | *Positive* | | 0.08 | 5 | 0.37 | 0.50 | 43 | 0.20 | 0.17 | 0.29 | 4.01 | 3.88 | 0 | 41 | -34 | 47 |
|  |  | |  |  | 0.03 | 0.06 | 119 | 0.01 | 0.20 | 0.29 | 3.96 | 3.83 | 0 | 36 | -16 | 50 |
|  |  | |  |  | 0.73 | 0.72 | 15 | 0.57 | 0.74 | 0.88 | 3.36 | 3.28 | 0.001 | 58 | -19 | -10 |
|  |  | |  |  | 0.70 | 0.72 | 17 | 0.53 | 0.81 | 0.89 | 3.27 | 3.2 | 0.001 | -48 | -13 | 33 |
|  |  | |  |  | 0.87 | 0.89 | 3 | 0.89 | 0.88 | 0.95 | 3.18 | 3.11 | 0.001 | 27 | -23 | 55 |
|  | *Negative* | | 0.90 | 1 | 0.76 | 0.64 | 12 | 0.64 | 0.68 | 0.51 | 3.41 | 3.33 | 0 | -22 | 15 | -23 |
| Empathic Distress | *Positive* | | 0.03 | 6 | 0.12 | 0.34 | 77 | 0.06 | 0.27 | 0.48 | 3.85 | 3.73 | 0 | 17 | -90 | 29 |
|  |  | |  |  | 0.65 | 0.89 | 21 | 0.46 | 0.42 | 0.55 | 3.67 | 3.57 | 0 | 28 | -65 | 48 |
|  |  | |  |  | 0.81 | 0.89 | 8 | 0.74 | 0.76 | 0.85 | 3.34 | 3.26 | 0.001 | -44 | 16 | 44 |
|  |  | |  |  | 0.87 | 0.89 | 3 | 0.89 | 0.83 | 0.85 | 3.26 | 3.18 | 0.001 | 31 | 3 | -47 |
|  |  | |  |  | 0.86 | 0.89 | 4 | 0.86 | 0.84 | 0.85 | 3.24 | 3.17 | 0.001 | -61 | -25 | 29 |
|  |  | |  |  | 0.87 | 0.89 | 3 | 0.89 | 0.85 | 0.85 | 3.22 | 3.15 | 0.001 | -63 | -25 | 0 |
|  | *Negative* | | No suprathreshold clusters | | | | | | | | | | | | | |

*Note.* Abbreviations: familywise error (FWE), correction (corr), false discovery rate (FDR

**Alternative anatomical region of interest approach**

While we utilised a coordinate-based region of interest (ROI) approach consistent with some prior literature (Eres et al., 2015), there are some limitations with this approach, including lack of alignment with anatomical boundaries. As such, we conducted supplementary analyses using anatomically defined ROIs. We utilised the 12 regions of interest from the Neuromorphometrics atlas (<http://www.neuromorphometrics.com/>). Maximum probability tissue labels were derived from the "MICCAI 2012 Grand Challenge and Workshop on Multi-Atlas Labeling". The volumes (mL) for each ROI per participant were calculated within the CAT12 software described in the main article. ROIs were chosen based on which ROIs encompassed the coordinates that formed the centre of the spherical ROIs used in the prior analyses. ROIs (listed in order of the matched coordinate ROI from Supplementary Table S1) were: triangular part of the inferior frontal gyrus right, frontal operculum left, anterior cingulate gyrus right, and left, supplementary motor cortex right, and left, precuneus right, and left, angular gyrus right, supramarginal gyrus left, superior frontal gyrus medial segment right and left. Regression models were run using R (R Core Team, 2021), to examine whether the empathy components (controlling for the covariates sex, age, TIV) were associated with each anatomical ROI. Correction for the total number of models run (12 ROIs x 4 empathy components = 48) was also computed.

Please see Supplementary Table S6 for the empathy components that were significantly associated with volumes of the anatomical ROIs. It was found that the smaller volumes of right and left precuneus were associated with higher affective sharing. Larger left frontal operculum volumes were associated with higher cognitive empathy. Finally, larger left frontal operculum volumes were also found to be associated with higher empathic distress. Only the left precuneus-affective sharing finding survived additional correction for the total numbers of models run.

**Supplementary Table S6**

*Regressions demonstrating associations between anatomical regions of interest volumes and empathy components*

| Empathy component | ROI | Estimate | Standard error | Statistic | P value |
| --- | --- | --- | --- | --- | --- |
| Affective sharing | Precuneus (r) | -0.07 | 0.02 | -2.63 | 0.0096* |
| Affective sharing | Precuneus (l) | -0.09 | 0.02 | -3.56 | 0.0005*† |
| Cognitive empathy | Frontal operculum (l) | 0.02 | 0.01 | 2.16 | 0.0327* |
| Empathic distress | Frontal operculum (l) | 0.01 | 0.01 | 2.14 | 0.0345* |

*Note*. Regions are from the Neuromorphometrics parcellation. * p < 0.05, † < 0.001 (survives correction for the total number of regressions run. Abbreviations: ROI = region of interest, r = right, l = left.

These findings using tanatomical ROIs support several of our findings using coordinate ROIs that were presented in the main paper. Both analyses found left precuneus negatively associated with affective sharing (anatomical analyses also detected right precuneus). We also found in both analyses that the frontal operculum/inferior frontal gyrus was positively associated with cognitive empathy. One finding using anatomical ROIs that was not previously detected was the positive association between the frontal operculum and empathic distress.

References

Eres, R., Decety, J., Louis, W. R., & Molenberghs, P. (2015). Individual differences in local gray matter density are associated with differences in affective and cognitive empathy. *NeuroImage*, *117*, 305–310. https://doi.org/10.1016/j.neuroimage.2015.05.038

Fan, Y., Duncan, N. W., de Greck, M., & Northoff, G. (2011). Is there a core neural network in empathy? An fMRI based quantitative meta-analysis. *Neuroscience & Biobehavioral Reviews*, *35*(3), 903–911. https://doi.org/10.1016/j.neubiorev.2010.10.009

R Core Team (2021). R: A language and environment for statistical computing. R Foundation for Statistical Computing, Vienna, Austria. URL https://www.R-project.org/.

Schurz, M., Radua, J., Aichhorn, M., Richlan, F., & Perner, J. (2014). Fractionating theory of mind: A meta-analysis of functional brain imaging studies. *Neuroscience & Biobehavioral Reviews*, *42*, 9–34. https://doi.org/10.1016/j.neubiorev.2014.01.009
